# Intake of slow-digesting carbohydrates is related to changes in the microbiome and its functional pathways in growing rats with obesity induced by diet

**DOI:** 10.3389/fnut.2022.992682

**Published:** 2022-11-30

**Authors:** Julio Plaza-Díaz, Manuel Manzano, Francisco Javier Ruiz-Ojeda, Maria D. Giron, Rafael Salto, Jose M. López-Pedrosa, Angela Santos-Fandila, Maria Teresa Garcia-Corcoles, Ricardo Rueda, Ángel Gil

**Affiliations:** ^1^Department of Biochemistry and Molecular Biology II, School of Pharmacy, University of Granada, Granada, Spain; ^2^Children’s Hospital of Eastern Ontario Research Institute, Ottawa, ON, Canada; ^3^Instituto de Investigación Biosanitaria de Granada (ibs.Granada), Complejo Hospitalario Universitario de Granada, Granada, Spain; ^4^Abbott Nutrition R&D, Granada, Spain; ^5^Institute of Nutrition and Food Technology “José Mataix”, Biomedical Research Centre, University of Granada, Granada, Spain; ^6^RG Adipocytes and Metabolism, Institute for Diabetes and Obesity, Helmholtz Diabetes Center at Helmholtz Center Munich, Munich, Germany; ^7^CIBER Physiopathology of Obesity and Nutrition, Instituto de Salud Carlos III, Madrid, Spain

**Keywords:** obesity, dietary carbohydrates, gastrointestinal microbiome, metabolism, microbiota, pediatric obesity, rats

## Abstract

**Introduction:**

The main cause of insulin resistance in childhood is obesity, which contributes to future comorbidities as in adults. Although high-calorie diets and lack of exercise contribute to metabolic disease development, food quality rather than the quantity of macronutrients is more important than food density. The purpose of the present study was to examine the effects of changing the quality of carbohydrates from rapidly to slowly digestible carbohydrates on the composition of the gut microbiota and the profiles of the functional pathways in growing rats with obesity due to a high-fat diet (HFD).

**Methods:**

During the course of 4 weeks, rats growing on an HFD-containing carbohydrates with different digestive rates were fed either HFD-containing carbohydrates with a rapid digestion rate (OBE group) or HFD-containing carbohydrates with a slow digestion rate (OBE-ISR group). A non-obese group (NOB) was included as a reference, and rats were fed on a rodent standard diet (AIN93G). An analysis of gut microbiota was conducted using 16S rRNA-based metagenomics; a linear mixed-effects model (LMM) was used to determine changes in abundance between baseline and 4 weeks of treatment, and functional pathways were identified. Gut microbiota composition at bacterial diversity and relative abundance, at phylum and genus levels, and functional profiles were analyzed by integrating the Integrated Microbial Genomes (IMG) database.

**Results:**

The groups showed comparable gut microbiota at baseline. At the end of the treatment, animals from the ISR group exhibited differences at the phylum levels by decreasing the diversity of Fisher’s index and *Firmicutes* (newly named as *Bacillota*), and increasing the Pielou’s evenness and *Bacteroidetes* (newly named as *Bacteroidota*); at the genus level by increasing *Alistipes, Bifidobacterium*, *Bacteroides*, *Butyricimonas*, *Lachnoclostridium*, *Flavonifractor*, *Ruminiclostridium 5*, and *Faecalibaculum* and decreasing *Muribaculum*, *Blautia*, and *Ruminiclostridium 9*. Remarkably, relative abundances of genera *Tyzzerella* and *Angelakisella* were higher in the OBE group compared to NOB and OBE-ISR groups. In addition, some microbiota carbohydrate metabolism pathways such as glycolysis, glucuronic acid degradation, pentose phosphate pathway, methanogenesis, and fatty acid biosynthesis exhibited increased activity in the OBE-ISR group after the treatment. Higher levels of acetate and propionate were found in the feces of the ISR group compared with the NOB and OBE groups.

**Conclusion:**

The results of this study demonstrate that replacing rapidly digestible carbohydrates with slowly digestible carbohydrates within an HFD improve the composition of the gut microbiota. Consequently, metabolic disturbances associated with obesity may be prevented.

## Introduction

Over the last century, non-communicable diseases (NCDs) have replaced communicable diseases as the primary cause of premature death worldwide, including heart disease, stroke, cancer, diabetes, and chronic lung disease. These diseases account for approximately 70% of all deaths worldwide (World Health Organization, WHO). NCDs and dysbiotic gut microbiota have been linked in several studies; therefore, establishing a causal association between dysbiotic microbiota and NCDs may represent a paradigm shift in the prevention and control of these diseases (1).

As a consequence of the obesity pandemic, insulin resistance, metabolic diseases, type 2 diabetes, non-alcoholic fatty liver disease (NAFLD), and certain cancers are at an increased risk (2). In this context, gut microbiota composition and its metabolites change in individuals with obesity, resulting in an increase of *Firmicutes* (newly named as *Bacillota*) to *Bacteroidetes* (newly named as *Bacteroidota*) ratio (3, 4), which is already established as an indicator of obesity and cardiometabolic disease in human and murine studies (5). Diet influences the human gut microbiota, and dietary interventions cause substantial changes in microbial diversity, both in the short and long term. These findings suggest that dietary interventions might improve the low microbial gene richness and the host metabolism of obese individuals (6).

A long-term high-fat diet (HFD) changes gut microbiota in experimental animals, such as rats and mice, leading to higher intestinal permeability and mucosal immune responses, contributing to obesity and chronic inflammation. As a result of increased permeability, lipopolysaccharide is translocated into the liver, kidney, and heart of obese rats. As a result, oxidative stress and lipid peroxidation are induced, and antioxidant enzyme activity is reduced. The structure of gut microbiota may be altered by HFD feeding, which may lead to the development of obesogenic bacteria and dysbiosis (7, 8). Furthermore, HFD increases the levels of phyla *Bacillota* and *Proteobacteria* (newly named as *Pseudomonadota*) and decreases the beneficial species of *Bifidobacterium* spp. and *Lactobacillus gasseri* (9). Indeed, HFD can induce gut microbial dysbiosis in more than 200 strains of mice with genetic variations (10), suggesting that dietary perturbations can lead to changes in gut microbiota despite variations in the host genome.

Generally, a metabolically healthy microbiota is mainly achieved by adhering to a diet that is low in animal fat, and animal protein, as well as fermentable dietary fiber. This serves as a growth substrate for bacteria and yeasts in the distal bowel. The opposite is true, and microbial dysbiosis is linked with a high-fat, high-protein diet, and sedentary lifestyle. Smoking, alcohol consumption, and relatively infrequent defecation are associated with a leaky mucosa, inflammation, and reduced production of short-chain fatty acids (SCFAs), including acetate and butyrate. In particular, dysbiotic microbiota is associated with a prolonged colonic transit time, which results in an increase in microbial proteolysis as a result of a shift in colonic metabolism. It has been reported that people who consume a high amount of protein and animal fat have a lower *Bacteroidota* to *Bacillota* ratio in their gut microbiota, whereas people who consume a high amount of fiber and carbohydrates (CHO) have a dominant genus of *Prevotella* (11).

Dietary fiber, in contrast to simple CHO, is not readily digested or absorbed by the host, but appears to affect the metabolic activities of intestinal bacteria or the interactions between bacteria and the host (12). In fact, slow-digesting CHO (ISR) influences endogenous substrate utilization during growth and development in pre-pubertal children, suggesting a beneficial effect on energy intake and CHO regulation/metabolism (13). Rats exhibit a higher abundance of *Bifidobacterium* spp. when inulin and fructooligosaccharide (FOS) are not hydrolyzed by mammalian enzymes but instead fermented by the bacterial community in the caecum and colon. A reduction in chronic intestinal inflammation was observed in both fructans, which reduced *Clostridium* cluster XI and *C. difficile* toxin gene expression (14). It has been shown that resistant maltodextrin is not digestible in small intestine and ferments in the colon. This stimulates the growth of probiotic microorganisms, particularly those belonging to the genera *Lactobacillus* and *Bifidobacterium*. As a result of this fermentation, the colon pH decreases, which in turn increases the amount of *Bifidobacterium* spp. found in the colon (15, 16).

According to the previous research, slow-digesting CHOs (ISR) provide a number of metabolic benefits during growth and development as well as a reduction in the predisposition to obesity later in life (17, 18). Recently, we have previously revealed that the replacement of rapid digestible CHO for slowly digestible CHO (ISR, containing isomaltulose, resistant maltodextrin, inulin, and FOS) in an HFD rat model prevented the diet-induced obesity-related adverse effects and improved lipid metabolism by different mechanisms in adipose tissue, muscle, and liver, which are related to modifications in key proteins, such as GLUT2 and GLUT4, FAS, SRBP1, AMPK, and Akt, among others (19). Currently, there are not enough basic studies analyzing microbial, metabolic, and inflammation data. Here, we aimed to evaluate the fecal microbiota and associated metabolic pathway changes after the consumption of those ISR in growing rats with HFD-induced obesity.

## Materials and methods

### Housing and experimental design

A total of thirty weanling male Wistar Han IGS (International Genetic Standard) rats (21–25 days of age) were provided by Charles Rivers (Orleans Cedex, France). Each animal was housed in a cage under a cycle of 12 h of light and 12 h of darkness. A temperature of 21°C was maintained in the room. The experimental procedures (approval code 29 October 2018/152) were carried out in accordance with the European Convention for the Protection of Vertebrate Animals used for Experimental and other Scientific Purposes (Directive 2010/63/EU) and to the Spanish National Research Council’s ethical guidelines for animal experimentation (RD 53/1 February 2013). Dietary obesity can be studied using an animal model induced by HFDs.

On the basis of the diet they received, rats (*n* = 10/group) were randomly assigned to three nutritional groups:

(i) The NOB group, a lean group of rats, was fed a standard rodent diet (AIN93G) (20).

(ii) The OBE group, an obese group of rats, was fed an HFD (21).

(iii) The rats in the OBE-ISR group were fed an HFD that included ISR.

The total fiber content of all diets was the same. In addition, both HFDs are isoenergetic. Table 1 contains all information regarding the diets. The nutritional intervention lasted for 4 weeks. Food and water were freely available to all rats, and their weight and food consumption were determined on a weekly basis. There has been previous publication on body composition, body fat mass, and lean body mass and metabolic parameters (19).

**TABLE 1:** Composition of diets.

Macronutrients	AIN93G	HFD	HFD- ISR
CHO *(g/100g diet)*	64.59	49.42	49.42
Sucrose *(g/100g CHO)*	14.37	36.07	
Isomaltulose *(g/100g CHO)*			26.40
Sucromalt^®^ *(g/100 g CHO)*			22.10
Cornstarch *(g/100g CHO)*	50.66	23.50	
Maltodextrins *(g/100 g CHO)*	18.97	24.43	34.50
Resistant maltodextrins *(g/100g CHO)*			10.00
Inulin:FOS *(g/100 g CHO)*			7.00
Cellulose *(g/100 g CHO)*	16.00	16.00	
*Total sugar (g/100 g CHO)*	*15.00*	*36.85*	*39.10*
*Total fiber (g/100 g CHO)*	*16.00*	*16.00*	*16.00*
*Glycemic load*	*726*	*687*	*338*
Protein *(g/100 g diet)*	17.00	24.19	24.19
Fat *(g/100 g diet)*	7.00	20.00	20.00
Energy *(calories/100 g diet)*	372.13	458.63	458.63

HFD, high-fat diet; RDC, diet with rapidly digestible CHO; ISR, diet with slowly digestible CHO; CHO, carbohydrates; Inulin:FOS, 1:1 mixture of fructooligosaccharides (FOS). Glycemic load is an estimation of the glycemic index of the carbohydrate blend resulting from the sum of each component’s glycemic index multiplied by its amount in the diet.

### Microbiota and related functional pathway profile analyses

A hermetically sealed, sterile container was used to collect fecal samples at the beginning and the end of the intervention. As soon as the samples were collected, they were immediately refrigerated after collection and kept at −80°C until they were used for analysis.

#### DNA extraction

A Stomacher-400 blender was used to homogenize the samples. As instructed by the manufacturer, DNA extraction was conducted using the QIAamp DNA Stool Mini Kit (QIAGEN, Barcelona, Spain), except for the incubation at 70°C. To ensure that gram-positive and gram-negative samples were lysed, the samples were mixed with the lysis buffer and incubated at 95°C. It was determined that the DNA yield can be calculated spectrophotometrically with a NanoDrop ND1000 spectrophotometer (Thermo Fisher Scientific, DE, United States) with absorbance ratios measured at A260/230 nm for salt and phenol contamination and A260/280 nm for protein contamination (22–24).

#### Sequencing

PCR was used to amplify the extracted DNA using the primers, 16S Amplicon PCR forward primer:

5′TCGTCGGCAGCGTCAGATGTGTATAAGAGACAGC CTACGGGNGGCWGCAG, and 16S Amplicon PCR Reverse Primer: 5′GTCTCGTGGGCTCGGAGATGTGTATAAGAGAC AGGACTACHV GGGTATCTAATCC targeting the V3 and V4 hypervariable regions of the bacterial 16S rRNA gene. PCRs were conducted in 25 μL reaction volumes of which 12.5 μL were 2X KAPA HiFi HotStart ready mix (KAPA Biosystems, Woburn, MA, United States). In addition, 5 μL of forward and reverse primers (1 μM) were prepared, as well as 2.5 μL of extracted DNA (10 ng). PCR cycling conditions were also identical for each PCR: initial denaturation at 95°C for 3 min, followed by cycles of denaturation at 95°C for 30 s, annealing at 55°C for 30 s, and elongation at 72°C for 30 s, with a final extension at 72°C for 5 min. As part of the PCR-clean up, AMPure XP beads (Beckman Coulter, Indianapolis, IN, United States) were employed to remove free primers and primer-dimer species from the 16S V3 and V4 amplicons. Immediately after this, the index PCR was performed at 95°C for 3 min. This was followed by 8 cycles at 95°C for 30 s, 55°C for 30 s, 72°C for 30 s, and 72°C for 5 min and held at 4°C. The Nextera XT Index Kit (Illumina, San Diego, CA, United States) was used to attach dual indices and Illumina sequencing adapters. Before quantification, AMPure XP beads (Beckman Coulter, Indianapolis, IN, United States) were employed to purify the pooled PCR products. Using a paired-end (2 × 300 nt) Illumina MiSeq sequencing system (Illumina, San Diego, CA, United States), the resultant amplicons were sequenced at MiSeq (Illumina, San Diego, CA, United States) (22–24).

#### Bioinformatic analysis

Illumina bcl2fastq2 Conversion Software v2.20 was used to demultiplex raw sequences, and raw data were imported into QIIME 2 2020.8 open-source software (25) using the q2-tools-import script which uses the PairedEndFastqManifestPhred33 input format. Sequencing of 16S rRNA genes was performed on V3-V4. Denoising was performed using DADA2 (26), which uses a quality-aware model of Illumina amplicon errors to obtain a distribution of sequence variances, each differing by one nucleotide. To truncate the forward reads at position 288 and trim them at position 6, the q2-dada2-denoise script was executed following the retrieving quality scores. We trimmed reverse reads at position 7 after truncating them at position 220. To remove chimeras, we applied the “consensus” filter, which detects chimeras in samples individually and removes those found in a sufficient fraction of samples. In addition, forward and reverse reads are merged during this step. Phylogenies were constructed with FASTTREE2 (*via* q2- phylogeny) (27) using all amplicon sequence variants (ASVs) aligned with MAFFT (28) *via* q2- alignment. To classify ASVs, a naïve Bayes taxonomy classifier was used (*via* q2-feature-classifier) (29) against the SILVA 16S V3-V4 v132_99 (30) along with a similarity threshold of 99%. As part of the data filtering process, samples with fewer than 10,000 reads were excluded. Both weighted (quantitative) and unweighted (qualitative) variants of UniFrac were determined, where the former accounts for the abundance of observed organisms, whereas the latter only accounts for their presence or absence.

#### Functional profiles

PICRUSt2 was used to predict potential functional profiles for sequenced samples (31). To summarize, phylotypes were arranged into a reference tree containing 20,000 full 16S rRNA genes from prokaryotic genomes in the Integrated Microbial Genomes (IMG) database. Clusters of Orthologous Groups of proteins (COG) and the Enzyme Commission numbers (EC) databases were used to annotate the functional characteristics of these genomes. EC numbers were first regrouped into MetaCyc reactions to infer MetaCyc pathways. Each pathway abundance was calculated as the harmonic mean of the key reaction abundances in each sample. Phylotypes abundances were corrected by 16S rRNA gene copy numbers and then multiplied by functional predictions to estimate the abundance of each gene family per sample.

### Quantitation of short-chain fatty acids

With some modifications, the method of Zeng and Cao (32) was used to analyze SCFAs in feces. In brief, a UPLC^®^ Acquity system from Waters (Milford, MA, United States) was coupled to a triple quadrupole mass spectrometer detector (XEVO-TQS) with electrospray ionization (ESI) interface in positive ion mode. Tandem mass spectrometers were operated in multiple reaction monitoring (MRM) mode with unit mass resolution set for Q1 and Q3 quadrupoles. Continuous infusion of standard solutions (1 mg L^–1^) optimized the mass spectrometric conditions for each compound. An electrospray ionization voltage of 2000 volts was applied to the capillary. In the cone, nitrogen was used as an auxiliary gas at 150 L h^–1^ and as a desolvation gas at 1000 L h^–1^. The source temperature was 150°C, and the desolvation temperature was 500°C. As a collision gas, argon (99.999% purity) was used at an approximate rate of 0.15 mL min^–1^.

Waters UPLC BEH C18 column (2.1 mm × 100 mm i.d., 1.7 μm particle size) was used for chromatographic analysis. The flow rate was 300 μL min^–1^, column temperature was 35°C, sample temperature was 8°C, and the injection volume was 1 μL. There was a gradient mobile phase consisting of 0.1% (v/v) of formic acid (FA) in water with 10 mM ammonium formate as solvent A and 0.1% (v/v) of FA in methanol:isopropanol (MeOH:IPA, 9:1 v/v) as solvent B. Gradient conditions were as follows: 0.0–4.0 min, 32% to 60% B; 4.0–4.8 min, 60% to 65% B; 4.8–4.9 min, 65% to 98% B; 4.9–5.5 min, 98% B and 5.5–6 min, 98% to 32% B for re-equilibration. The total run time was 6 min. The weak solvent was a mixture of 30% of water and 70% of acetonitrile (MeCN), and the strong solvent was 100% MeOH.

For instrument control and data acquisition and analysis, MassLynx software version 4.1 was used. With only a few changes in dilutions, samples and standards were processed according to the protocol described in the method of Zheng et al. (32). The procedure involves derivatization with 0.1 M of O-Benzylhydroxylamine hydrochloride (BHA) in MeOH and 0.25 M of N-(3-Dimethylaminopropyl)-N’-ethylcarbodiimide hydrochloride (EDC) in MeOH at 25°C for 1 h. A 50% methanol solution in water was used to dilute the samples after incubation, and dichloromethane was used to extract them. A specific volume of 50% aqueous MeOH was added after the extract was evaporated to rehydrate the residual. to inject samples into the UPLC-MS/MS system, samples were collected in vials. In SCFA analysis, specific commercial standards are used to quantify each compound individually with its corresponding standard: acetic acid (AA) ref: 71251-5ML; propionic acid (PA) ref: 94425-5ML; iso-butyric acid (Iso-BA) ref: 46935-U-500MG; butyric acid (BA) ref: 19215-5ML; 2-methyl-butyric acid (2M-BA) ref: 49659-1ML; iso-valeric acid (Iso-VA) ref: 78651-5ML; valeric acid (VA) ref: 75054-5ML; 3-methyl-valeric acid (3M-VA) ref: 222453-5G; 4-methyl-valeric acid (4M-VA) ref: 277827-25G; hexanoic acid (HA) ref: 21529-5ML; all of them are from Sigma-Aldrich. In addition, internal standards have also been used to improve accuracy and precision: sodium acetate-d3 (d3-AA) ref: 176079-25G; propionic acid-d5 (d5-PA) ref: 596507-1G; butyric acid-d7 (d7-BA) ref: 488399-5G; hexanoic acid-d11 (d11-HA) ref: 448168-1G; they also are from Sigma-Aldrich. Due to the lack of internal standards in all the studied compounds, some of them were used when the compounds had similar structures. The calibration curve was done in a linear range of 0.0005–1.034 ug/ml for feces samples.

### Determination of serum bile acid profiles

Metabolite profiles were determined as described previously (33, 34). In brief, OWL Metabolomics (Derio, Spain) assessed bile acids on an established rodent serum platform. A UHPLC-time-of-flight (TOF)–Mass spectroscopy (MS)-based platform (Agilent Technologies, Santa Clara, CA, United States) was used here to semi-quantify bile acid species in methanol serum extracts. The TargetLynx application manager for MassLynx 4.1 (Waters Corp., Milford, MA, United States) was used to handle the data obtained with the aforementioned platform. The data obtained during the process were processed with R software v3.2.0 (19).

### Biochemical parameters

In this study, serum interleukin-(IL)-1β, monocyte attractant proteins 1 (MCP-1), leptin, and tumor necrosis factor-alpha (TNF-α) were measured using Bio-Plex 200 system (Bio-Rad, Hercules, CA, United States). Serum glucagon-like peptide-1 (GLP-1) was measured using an ELISA kit (Mercodia, Uppsala, Sweden) according to the manufacturer’s instructions (19).

### Statistical analysis

Median and range are used to express data. To determine differences due to intervention time and treatment, we used a general linear model for repeated measures. For time and treatment × time, *p*-Values were calculated with LSD *post hoc* multiple comparisons with different letters indicating significant differences (*p* < 0.05).

Data regarding functional pathways profiles and SCFAs are presented as a mean and standard mean error (SEM). A statistically significant difference was considered to be *p* < 0.05. In the case of variables that were not normally distributed, they were log-transformed, and/or outliers were removed (without achieving a loss of value from samples of up to 15%). To ensure a clear understanding of the data, the values are presented untransformed. To assess differences at baseline in the relative abundance of bacteria (phylum and genus), as well as for the alpha indexes and beta diversity, the Mann–Whitney *U* test was applied. When testing differences in beta diversity, we used ADONIS-2 function from the vegan package using 10,000 permutations for calculating *p*-Values.

The Kruskal–Wallis test was used to analyze the differential relative abundance of general metabolic pathways. To correct for multiple comparisons, the Benjamini–Hochberg correction (FDR) was applied. GraphPad Prism 8 was used to create all metabolic pathway images. Unless otherwise specified in the figure legend, data are presented as mean ± SEM. As indicated in the respective figure legend, statistical significance was determined by a one-way ANOVA, followed by Tukey’s multiple comparison test. Differences reached statistical significance with *p* < 0.05. At the end of the intervention, Pearson’s correlations were used to examine relationships between diversity indices, microbiome variables, anthropometric, inflammation, metabolic parameters, serum bile acids, and fecal SCFA levels. Multiple testing was corrected by applying the FDR procedure (35). Using the corrplot function in R studio, associations were expressed. The graph shows only significant and corrected associations (36). Correlation values were shown within the graphs in red and blue colors, the negatively correlated in red (−1) and the positively correlated in blue (+1).

## Results

**FIGURE 1:**
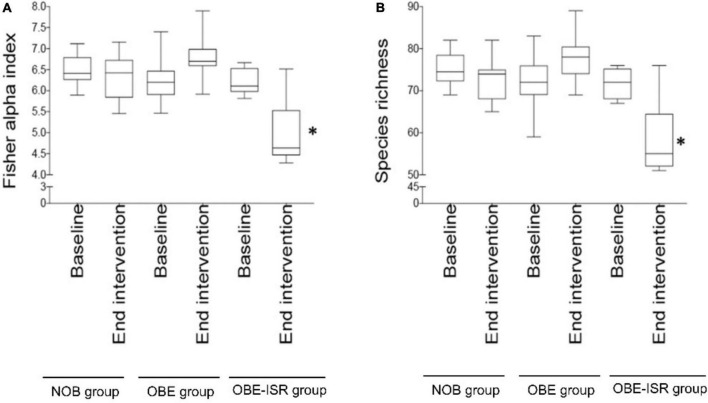
Diversity indices across the intervention. **(A)** Fisher’s alpha index abundance in the non-obese (NOB), obese (OBE), and obese with slowly digestible CHO (OBE-ISR) groups at the baseline and at the end of intervention after 4 weeks. **(B)** Species richness abundance in the non-obese (NOB), obese (OBE), and obese with slowly digestible CHO (OBE-ISR) groups at the baseline and at the end of intervention after 4 weeks. *p < 0.05.

**FIGURE 2:**
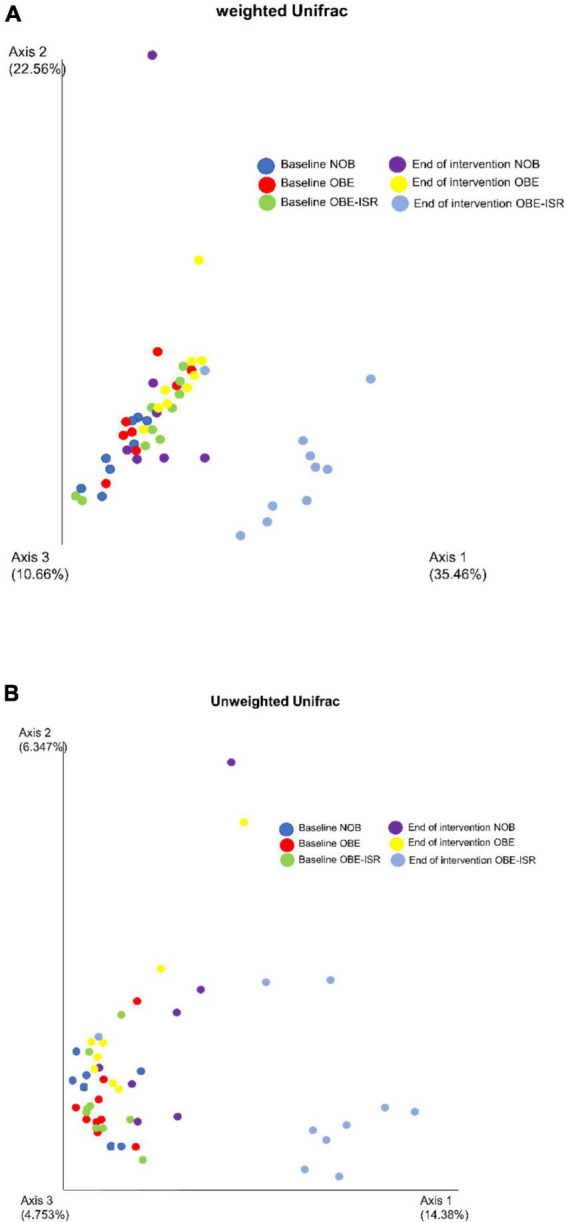
**(A)** Weighted UniFrac distances (ADONIS *p* = 0.001, *r*^2^ = 0.495). **(B)** Unweighted UniFrac distances (ADONIS *p* = 0.001, *r*^2^ = 0.429). Beta diversity is represented by principal coordinate analysis (PCoA) based on unweighted UniFrac distance.

In this study, we observed the replacement of rapidly digestible CHO for ISR within an HFD-promoted change in the gut microbiota composition. Table 2 represents the phylum-based abundances of *Verrucomicrobia* (newly named as *Verrucomicrobiota*), *Pseudomonadota, Bacillota, Deferribacteres* (newly named as *Deferribacterota*), *Bacteroidota*, and *Actinobacteria* (newly named as *Actinobacteriota*) in the NOB, OBE, and OBE-ISR at the baseline and the end of the treatment. Indeed, the OBE-ISR group showed significant changes in *Bacteroidota*, *Actinobacteriota, Bacillota*, and *Pseudomonadota.* At the phylum level, the relative abundance was similar to groups at the baseline. Based on the interaction *time* × *treatment* after 4 weeks, bacteria from the OBE-ISR group showed a decrease in Fisher’s index (*p* = 0.004) and *Bacillota* (*p* = 0.024) and an increase in Pielou’s evenness (*p* = 0.004) and *Bacteroidota* (*p* = 0.002) (Table 2). At the genus level, the groups had comparable gut microbiota at baseline. After 4 weeks of treatment, OBE and OBE-ISR groups had a higher proportion of sequences in the *Alistipes* genus than NOB group (*p* = 0.023). The OBE-ISR group exhibited an increase in the relative abundances of the genera *Bifidobacterium* (*p* = 0.045), *Bacteroides* (*p* = 0.001), *Butyricimonas* (*p* = 0.023), *cc* (*p* < 0.001), *Flavonifractor* (*p* = 0.003), *Ruminiclostridium 5* (*p* = 0.039), and *Faecalibaculum* (*p* = 0.009). On the contrary, the OBE-ISR group revealed a decrease in the relative abundances of the genera *Muribaculum* (*p* = 0.01), *Blautia* (*p* = 0.024), and *Ruminiclostridium 9* (*p* = 0.002). Interestingly, relative abundances of genera *Tyzzerella* (*p* = 0.006) and *Angelakisella* (*p* = 0.003) increased in the OBE group compared to NOB and OBE-ISR groups, and the OBE-ISR group exhibited similar levels to NOB group at the end of the intervention (Table 3). Remarkably, we observed that both Fisher’s alpha and species richness abundance in the OBE-ISR group were reduced compared to NOB and OBE groups at the end of intervention after 4 weeks (Figure 1 and Table 4). The visualization of weighted (ADONIS *p* = 0.001, *r*^2^ = 0.495) and unweighted (ADONIS *p* = 0.001, *r*^2^ = 0.429) UniFrac distance data demonstrated that fecal samples from the OBE-ISR group after 4 weeks of nutritional intervention were separated from those of NOB and OBE groups (Figure 2).

**TABLE 2:** Relative abundances at the phylum level.

	Baseline			End of intervention			*p*-Values	
Variables	NOB	OBE	OBE-ISR	NOB	OBE	OBE-ISR	Time (t)	Treatment × t
Sequences	248,591 (178,631−275,608)	229,492 (132,581−301,851)	216,060 (162,561−316,366)	230,278 (140,582−448,367)	206,946 (126,659−278,218)	189,699 (146,887−259,876)	0.691	0.262
*Actinobacteriota*	0.98 (0.15−0.88)	0.46 (0.09−0.93)	0.92 (0.4−2.58)	0.28 (0.08−1.63)	0.07 (0−0.8)	1.81 (0.06−5.26)	0.843	0.088
*Bacteroidota*	30.31 (15.26−38.66)	35.99 (30.3−45.41)	37.32 (14.01−45.01)	34.24 (13.09−38.76)^a^	30.63 (17.42−37.97)^a^	44.26 (38−55.37)^b^	0.384	0.002
*Cyanobacteriota*	0 (0− 0.12)	0 (0.0.02)	0 (0−0)	0 (0−0.30)	0 (0−0.04)	0 (0−0)	0.511	0.734
*Deferribacterota*	0.25 (0.04−0.62)	0.13 (0.04−1.46)	0.08 (0−0.32)	0.03 (0−0.15)	0.34 (0−1.04)	0 (0−1.42)	0.758	0.529
*Bacillota*	26.32 (25.77−30.47)	27.88 (23.12−37.46)	26.75 (21.85−32.24)	25.78 (20.72−78.16)^a^	35.75 (30.26−73.4)^a^	20.14 (15.82−30.74)^b^	0.122	0.024
*Fusobacteriota*	0 (0−0)	0 (0−0)	0 (0−0)	0 (0−0)	0 (0−0)	0 (0−0)	−	−
*Candidatus Patescibacteria*	0 (0−0.2)	0 (0−0.01)	0 (0−0.16)	0 (0−0.03)	0 (0−0)	0 (0−0)	0.384	0.486
*Pseudomonadota*	6.08 (5.08−8.56)	5.38 (4.72−8.7)	7.4 (5.41−8.63)	4.23 (0.58−5.31)^a^	5.25 (1.78−6.34)^a^	6.22 (4.61−8.96)^b^	<0.001	0.144
*Mycoplasmatota*	0 (0−0.3)	0 (0−0.05)	0 (0−0)	0 (0−0)	0.02 (0−0.04)	0 (0−0.02)	0.524	0.182
*Verrucomicrobiota*	34.57 (27.64−51.02)	31.38 (16.68−36.91)	27.37 (16.5−50.84)	31.77 (2.65−39.74)	24.05 (6.65−34−83)	24.4 (8−39.82)	0.143	0.968
Unassigned	0.02 (0−0.05)	0.01 (0−0.05)	0.02 (0−0.05)	0.03 (0−3.59)	0.03 (0.01−0.57)	0.02 (0−0.05)	0.18	0.332

Data are expressed as median and range. A general linear model for repeated measures was used to determine differences. *p*-Values were determined for time and treatment × time, and different letters mean significant differences (*p* < 0.05) and were calculated with LSD *post hoc* multiple comparisons for observed means. In light of the updated taxonomic designations for the taxonomic of *Actinobacteria, Bacteroidetes, Cyanobacteria, Deferribacteres, Firmicutes, Fusobacteria, Proteobacteria, Tenericutes*, and *Verrucomicrobia*, we refer to them as *Actinobacteriota, Bacteroidota, Cyanobacteriota, Deferribacterota, Bacillota, Fusobacteriota, Pseudomonadota, Mycoplasmatota*, and *Verrucomicrobiota*, respectively.

**TABLE 3:** Relative abundances at the genus level.

	Baseline			End of intervention			*p*-Values	
Variables	NOB	OBE	OBE-ISR	NOB	OBE	OBE-ISR	time (t)	Treatment × t
*Bifidobacterium*	0.66 (0.14−1.22)	0.39 (0−0.8)	0.67 (0.23−2.23)	0.08 (0−0.18)^a^	0.01 (0−0.39)^a^	1.25 (0−2.54)^b^	0.058	0.045
*Enterorhabdus*	0.1 (0.01−0.18)	0.5 (0−0.1)	0.09 (0−0.21)	0.17 (0.02−1.63)^a^	0.04 (0−0.2)^b^	0 (0−0.15)^b^	0.14	0.073
*Bacteroides*	6.18 (1.69−11.04)	9.2 (6.69−11.93)	10.55 (2.35−13.29)	9.82 (2.05−16.54)^a^	8.79 (4.71−11.27)^a^	18.61 (10.58−23.02)^b^	0.001	0.001
*Butyricimonas*	0.9 (0.1−0.37)	0.14 (0.01−0.31)	0.11 (0.02−0.3)	0.19 (0−1.9)^a^	0.31 (0.07−1.04)^a^	1.21 (0.42−8.05)^b^	0.004	0.023
*Muribaculum*	6.62 (3.63−12.33)	7.4 (5.55−9.69)	7.36 (3.11−9.62)	5.16 (0.77−6.38)^a^	2.56 (1.08−3.43)^ab^	0.89 (0.17−2.75)^b^	<0.001	0.01
*Alistipes*	3.68 (2.64−7.96)	6.04 (3.13−8.86)	3.82 (1.68−8.3)	3.02 (1.51−5.76)^a^	5.46 (3.63−7.1)^b^	5.77 (2.82−10.18)^b^	0.896	0.023
*Rikenellaceae* RC9 gut group	1.07 (0.5−2.46)	1.49 (0−3.55)	0.51 (0−3.17)	2.03 (1.66−7.43)^a^	4.58 (1.63−8.89)^b^	0 (0−6.56)^c^	0.001	0.01
*Parabacteroides*	3.40 (0.67−4.91)	3.24 (2.49−7.41)	4.16 (1.08−6.63)	1.8 (0.57−7.43)^a^	4.31 (2.14−6.9)^a^	16.07 (8.31−18.88)^b^	<0.001	
*Mucispirillum*	0.25 (0.04−0.62)	0.13 (0.04−1.46)	0.08 (0−0.32)	0.03 (0−0.15)	0.34 (0−1.04)	0 (0−1.42)	0.758	0.529
*Lactobacillus*	5.97 (0.31−13.48)	3.43 (0.73−10.96)	3.08 (1.2−0.78)	0.08 (0.03−1.33)	0.11 (0.02−0.77)	0.43 (0.01−1.06)	<0.001	0.646
*Clostridium* sensu stricto 1	0.39 (0.12−0.88)	0.47 (0.17−1.41)	0.71 (0−1.89)	0.19 (0−1.55)	0.08 (0−1−21)	0 (0−0.92)	0.027	0.201
*Blautia*	0.86 (0.28−3.49)	1.47 (0.09−4.22)	1.31 (0.37−2.89)	1.02 (0.1−3.73)^a^	3.98 (1.04−10.86)^b^	0.71 (0.09−5.48)^a^	0.124	0.024
*Lachnoclostridium*	0.14 (0.08−0.37)	0.1 (0−0.37)	0.22 (0.05−0.51)	0.19 (0.06−1.04)^a^	0.05 (0−0.89)^a^	4.39 (0.02−7.59)^b^	<0.001	
*Lachnospiraceae* NK4A136 group	0.08 (0−0.2)	0.15 (0−0.35)	0.02 (0−0.06)	0.13 (0−0.87)^ab^	0.29 (0−2.48)^b^	0 (0−0.67)^a^	0.105	0.517
*Lachnospiraceae* UCG-004	0.10 (0−0.35)	0.09 (0−0.36)	0.18 (0.01−0.44)	0.01 (0−0.38)	0.07 (0−0.33)	0 (0−0.09)	0.078	0.169
*Tyzzerella*	0.6 (0.07−0.89)	0.55 (0.06−1.19)	0.67 (0−0.99)	0 (0−0.01)^a^	0.84 (0.55−1.57)^b^	0 (0−0.82)^a^	0.006	
*Anaerotruncus*	0.91 (0.11−1.56)	0.78 (0.03−1.38)	0.53 (0.16−1.67)	0.58 (0.21−0.95)^a^	0.53 (0.23−2.06)^ab^	0.01 (0−1.05)^b^	0.022	0.19
*Angelakisella*	0.09 (0−0.12)	0.08 (0.02−0.16)	0.06 (0.03−0.14)	0.02 (0−0−13)^a^	0.17 (0.08−0.31)^b^	0 (0−0.14)^a^	0.891	0.003
*Flavonifractor*	0.07 (0−0.29)	0.18 (0−0.77)	0.14 (0−0.53)	0.47 (0.07−1.21)^ab^	0.1 (0−0.83)^a^	1.16 (0.17−1.57)^b^	<0.001	0.003
*Oscillibacter*	1.67 (0.89−2.8)	1.89 (0.52−3.63)	1.33 (0.79−2.74)	1.91 (1.39−3.72)	2.77 (2.04−4.11)	3.82 (0.03−6)	0.018	0.314
*Ruminiclostridium* 5	0.05 (0−0.21)	0.08 (0−0.18)	0.04 (0.02−0.13)	0.24 (0.11−0.67)^ab^	0.08 (0−1.08)^a^	0.79 (0−2.27)^b^	0.001	0.039
*Ruminiclostridium* 9	1.39 (0.76−4.06)	2.81 (1.37−6.6)	2.19 (0.98−3.53)	2.68 (1.04−6.13)^a^	6.33 (3.03−17.71)^b^	0 (0−2.36)^a^	0.111	0.002
*Ruminococcaceae* UCG-014	0.18 (0−1.13)	0.26 (0.03−0.84)	0.08 (0−1.13)	0.26 (0.06−2.12)	0.51 (0.21−1.9)	0.36 (0−1.74)	0.059	0.81
*Eubacterium coprostanoligenes* group	0.73 (0.21−1.88)	0.84 (0.2−1.85)	1.23 (0.14−2.43)	0.92 (0.39−1.06)	0.24 (0.02−0.87)	0.41 (0.01−1.85)	0.011	0.11
*Erysipelatoclostridium*	0.66 (0.1−1.92)	0.77 (0.36−1.25)	0.62 (0.35−3.7)	0.13 (0.02−59.4)	1.05 (0.48−3.09)	0.2 (0−0.5)	0.276	0.271
*Faecalibaculum*	0.01 (0−0.04)	0.01 (0−0.05)	0.04 (0−0.35)	0 (0−0.03)^a^	0 (0−0.02)^a^	0.08 (0−0.5)^b^	0.19	0.009
*Faecalitalea*	0 (0−0)	0 (0−0)	0 (0−0)	0.35 (0−1.4)	0.97 (0.65−1.73)	0.48 (0−2.93)	<0.001	0.263
*Turicibacter*	0.21 (0.05−0.86)	0.32 (0.14−0−52)	0.16 (0.06−0.76)	0 (0−0.55)	0 (0−0.13)	0.04 (0−0.49)	<0.001	0.378

Data are expressed as median and range. A general linear model for repeated measures was used to determine differences. *p*-Values were determined for time and treatment × time, and different letters mean significant differences (*p* < 0.05) and were calculated with LSD *post hoc* multiple comparisons for observed means.

**TABLE 4:** Alpha indexes.

	Baseline			End of intervention			*p*-Values	
Variables	NOB	OBE	OBE-ISR	NOB	OBE	OBE-ISR	Time (t)	Treatment × t
Shannon	1.60 (1.57−1.62)	1.60 (1.57−1.63)	1.61 (1.60−1.65)	1.61 (1.34−1.67)	1.60 (1.58−1.67)	1.62 (1.59−1.64)	0.999	0.834
Inverse Simpson	4.29 (4.14−4.42)	4.29 (3.29−4.38)	4.33 (4.25−4.43)	4.31 (2.90−4.66)	4.24 (4.13−4.57)	4.52 (4.27−4.58)	0.307	0.533
Pielou’s evenness	0.37 (0.36−0.38)	0.38 (0.36−0.38)	0.38 (0.37−0.39)	0.38 (0.32−0.39)^a^	0.37 (0.36−0.39)^a^	0.40 (0.37−0.41)^b^	0.102	0.004
Simpson	0.77 (0.76−0.78)	0.77 (0.70−0.77)	0.77 (0.76−0.77)	0.77 (0.65−0.78)	0.76 (0.76−0.78)	0.78 (0.76−0.78)	0.632	0.403

Data are expressed as median and range. A general linear model for repeated measures was used to determine differences. *p*-Values were determined for time and treatment × time, and different letters mean significant differences (*p* < 0.05) and were calculated with LSD *post hoc* multiple comparisons for observed means.

**FIGURE 3:**
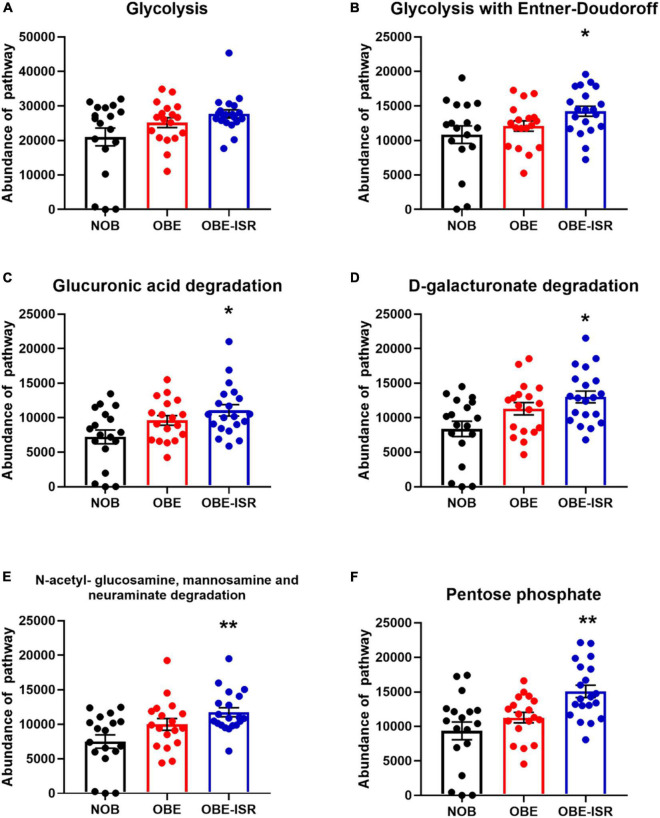
Major metabolic pathway categories relative to the abundance of each sample, including non-obese (NOB), obese (OBE), and experimental treatment slow-digesting carbohydrates OBE-ISR. **(A)** Glycolysis: Glycolysis I (from glucose-6-phosphate). **(B)** Glycolysis with Entner–Doudoroff: superpathway of glycolysis and the Entner–Doudoroff pathway. **(C)** Glucuronic acid degradation: Glucurocat pathway. **(D)** D-galacturonate degradation II: Galacturocat-pwy. **(E)** N-acetylglucosamine, mannosamine, and neuraminate degradation: Glcmannanaut-pwy, superpathway of N-acetylglucosamine, N-acetylmannosamine, and N-acetylneuraminate degradation. **(F)** Pentose phosphate pathway. *p < 0.05; **p < 0.01.

**FIGURE 4:**
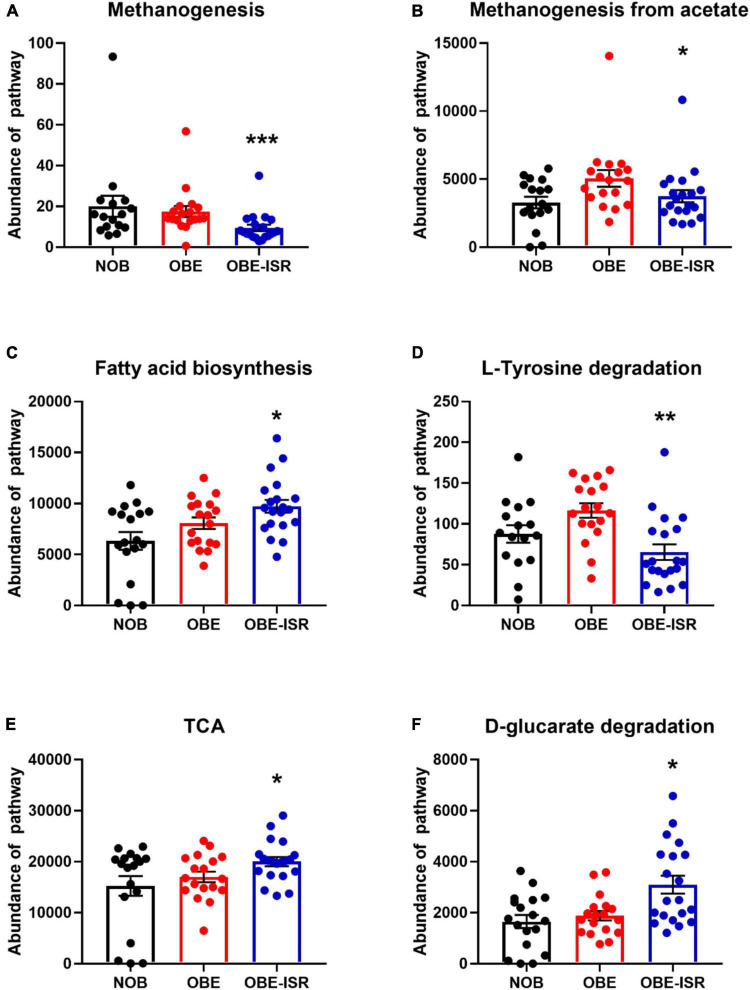
Major metabolic pathway categories relative to the abundance of each sample, including non-obese (NOB), obese (OBE), and experimental treatment of obese with slow-digesting carbohydrates OBE-ISR. **(A)** Methanogenesis-pwy, superpathway of methanogenesis. **(B)** Methanogenesis from acetate: Meth-acetate-pwy. **(C)** Fatty acid biosynthesis: superpathway of fatty acid biosynthesis initiation (*Escherichia coli*). **(D)** L-tyrosine degradation I: TYRFUMCAT-PWY. **(E)** TCA: Tricarboxylic acid cycle (prokaryotic). **(F)** D-glucarate degradation I. *p < 0.05; **p < 0.01.

At the end of the experiment, we observed significant increases in the activity of the different metabolic pathways in the OBE-ISR group, including glycolysis with the Entner–Doudoroff pathway, glucuronic acid degradation, D-galacturonate degradation, the super pathway of N-acetylglucosamine, N-acetylmannosamine and N-acetylneuraminate degradation, and the pentose phosphate pathway (PPP) (Figure 3). Moreover, key metabolic pathways such as methanogenesis, methanogenesis from acetate, fatty acid biosynthesis pathway, L-tyrosine degradation, D-glucarate, and tricarboxylic acid cycle (TCA) degradation were significantly enhanced in the more abundant bacteria isolated from the ISR-treated group after 4 weeks of the treatment (Figure 4).

In addition, we evaluated the fecal levels of SCFAs in these experimental groups. We observed that the OBE-ISR group had the highest levels of acetate and propionate, being statistically different from the other two groups NOB and OBE. However, no differences in butyrate levels were observed (Table 5). In addition, Pearson’s correlations between anthropometric, physiological, inflammatory and metabolic traits, and bacterial variables and serum levels of bile acids revealed some associations in NOB, OBE, and OBE-ISR groups (Supplementary Tables 1–3).

**TABLE 5:** Short-chain fatty acid (SCFA) production of experimental groups fed on the different experimental diets for 4 weeks to induce obesity.

	NOB	OBE	OBE-ISR
Acetic acid *(*μ*g/g dry feces)*	1292.0 ± 335.7^a^	609.7 ± 134.2^a^	3388.0 ± 670.7^b^
Propionic acid *(*μ*g/g dry feces)*	785.3 ± 218.2^a^	529.1 ± 159.8^a^	1604.0 ± 256.6^b^
Butyric acid *(*μ*g/g dry feces)*	351.9 ± 81.1^a^	478.3 ± 125.8^a^	618.8 ± 110.1^a^

Short-chain fatty acids data expressed as mean ± SEM. Different small letters indicate the significant differences (*p* < 0.05) among groups as shown analysis of variance (ANOVA). Data of gut microbiota are given as median and range. Different small letters indicate the significant differences (*p* < 0.05) among groups, as shown by the Kruskal–Wallis *H* test corrected by the Bonferroni *post hoc* test.

Body weight was negatively correlated with *Butyricimonas* and *Ruminiclostridium* 5 in the NOB group. In the case of primary bile acids, which are taurocholic acid and glycocholic acid (derivatives of cholic acid) and taurochenodeoxycholic acid and glycochenodeoxycholic acid (derivatives of chenodeoxycholic acid) (37), and SCFA, in the NOB group, we observed that chenodeoxycholic acid exhibited a positive association with *Muribaculum*; glycocholic acid was positively correlated with *Bifidobacterium*; glycochenodeoxycholic acid was negatively correlated with *Blautia*; and *Ruminiclostridium* 5 was positively associated with taurocholic and taurodeoxycholic acids. Furthermore, TG (50:1, glycerolipids. Subclass: Triacylglycerols). Composition: TG (16:0 + 18:1 + 16:0) was negatively associated with *Bacteroidota*. PE (16:0/20:4, glycerophospholipids. Subclass 1-ether, 2-acylglycerophosphoethanolamine) was negatively correlated with the *Rikenellaceae* RC9 gut group, and acetic and propionic acid levels showed a negative association with methanogenesis and L-tyrosine degradation. Finally, acetic acid was positively associated with *Butyricimonas* (Supplementary Table 1). On the contrary, we found that GLP-1 and IL-1β were positively correlated with the *Rikenellaceae* RC9 gut group and *Blautia* in the NOB group, respectively. *Bacteroides* exhibited a negative correlation with glycolysis with the Entner–Doudoroff pathway, glucuronic acid degradation, D-galacturonate degradation, the super pathway of N-acetylglucosamine, N-acetylmannosamine and N-acetylneuraminate degradation, and PPP. In the same way, *Flavonifractor* was negatively associated with D-galacturonate degradation, methanogenesis from acetate, and the fatty acid biosynthesis pathway (Supplementary Table 1).

The OBE group revealed that taurodeoxycholic acid was negatively associated with *Butyricimonas* and positively associated with GLP-1 levels. Besides, *Blautia* showed a negative association with D-glucarate degradation (Supplementary Table 2).

In the case of the OBE-ISR group, we have observed that cholic acid exhibited a positive association with *Faecalibaculum* and glucuronic acid degradation, and chenodeoxycholic acid with glucuronic acid degradation, PPP, and *Muribaculum*. Ursodeoxycholic and hyodeoxycholic acids were highly correlated with glucuronic acid degradation, *Bacteroidota*, *Muribaculum*, and *Faecalibaculum.* Glycocholic acid was positively associated with methanogenesis, methanogenesis from acetate, and L-tyrosine degradation. Moreover, deoxycholic acid was positively associated with *Butyricimonas, Ruminiclostridium* 5, and *Faecalibaculum.* Glycochenodeoxycholic acid was positively correlated with methanogenesis. *Bacteroidota* showed a positive association with taurocholic and taurodeoxycholic acids, and the last one also with glucuronic acid degradation. PE (16:0/20:4) was correlated negatively with *Bifidobacterium*, *Butyricimonas*, and *Faecalibaculum.* Butyric acid was positively associated with *Ruminiclostridium* 5. On the contrary, acetic and propionic acids showed a negative association with TCA (Supplementary Table 3).

We also found that glucagon-like peptide-1 (GLP-1) was negatively associated with gut microbiota *Bacteroides* whereas TNF-α and IL-1β were positively correlated with *Ruminiclostridium* 5 in the ISR group. In addition, *Bacillota* and *Ruminiclostridium* 9 were positively related to the super pathway of N-acetylglucosamine, N-acetylmannosamine, and N-acetylneuraminate degradation, as well as *Bacillota* with galacturonate degradation and PPP. Finally, *Flavonifractor* was negatively correlated with L-tyrosine degradation in the ISR group (Supplementary Table 3).

## Discussion

It has been proposed that gut microbiota is an integral component of human health. However, there is little information available regarding the composition of the gut microbiota during childhood. In spite of the suggestion that the microbiota reaches a relatively stable state in the first 3 years of life, other evidence indicates that it continues to develop during adolescence (38). In several studies, gut microbes and their hosts have been shown to interact metabolically, and dietary factors have a direct impact on the composition of the gut microbiota, which is thought to regulate systemic metabolism (39). For the development and growth of a child, CHO is one of the most significant sources of energy (40). Nonetheless, the quality of CHO is crucial for optimal glycemic control, insulin response, and weight management (13, 41, 42). In that context, we have previously reported that the OBE-ISR group improves insulin sensitivity and reduces dyslipidemia in growing rats, as well as GLP-1 augmented levels (19). Using a well-established HFD rodent model of childhood diet-induced obesity, we evaluated the effects of a specialized CHO diet on the gut microbiota composition by comparing its quality rather than its quantity (rapid digestible vs. slowly digestible CHO). As demonstrated here, the consumption of ISR within an HFD improved the composition of the gut microbiota as well as the functional pathway profile in obesity-growing rats, thereby potentially protecting against the development of metabolic disturbances.

Inflammation and decreased insulin sensitivity are linked with obesity and changes in fecal microbiota (43). Studies suggest that maintaining or restoring a healthy gut ecosystem may help to prevent the early onset of obesity, from a young age (44). There is evidence that insulin resistance in children is connected with a number of *Pseudomonadota* species, as well as a lower abundance of gram-negative bacteria and bacteria involved with butyrate production (45). It has been hypothesized that obese children have significantly higher *Bacillota* and lower *Bifidobacterium* than lean children (46). However, a meta-analysis of the gut microbiome revealed no correlation between these two phyla and obesity (47). As a result, there is a tendency for less bacterial diversity (4) and a reduced abundance of *Prevotella* (48). Both adults and children are affected by obesity, which results in changes in microbial diversity. In this matter, the causal relationship is still controversial; however, it is well-known that weight loss in people with obesity recovers gut microbiota composition toward a healthy status or similar to lean individuals (49).

Children who consume a Western-style diet have a more prevalent presence of *Bacteroidaceae* and *Ruminococcaceae*, as well as a lower presence of *Prevotellaceae* (50). The composition of the gut microbiota has been influenced by the consumption of certain types of fiber since early childhood. Human milk oligosaccharides and prebiotics added to infant formula are bifidogenic and inhibit pathogen growth. In addition, inulin increases *Bifidobacterium* abundance, while dietary fiber decreases fecal pH and increases alpha diversity and calcium absorption (50).

The use of rats after weaning may be able to mimic childhood conditions. After weaning, rats given an HFD developed many of the characteristics of the metabolic syndrome such as central obesity, systolic and diastolic hypertension, altered fasting glucose levels, hypertriglyceridemia, and decreased HDL cholesterol levels (51). During the present study, the nutritional intervention lasted for 4 weeks from weaning to infancy. Here, we observed that rats fed HFD supplemented with the ISR exhibited significant changes in *Bacteroidota, Actinobacteriota*, *Bacillota*, and *Pseudomonadota*, indicating an improvement of the gut microbiota profile disturbed by rapid digestible CHO/HFD-induced obesity. Indeed, obesity *per se* drives a lower relative abundance of *Bacteroidota* and reduced biodiversity in the gut microbiota (52). The interaction *time* × *treatment* showed that after 4 weeks of treatment, bacteria in the OBE-ISR group showed a decrease in *Bacillota* abundance, a decrease in Fisher’s index, and an increase in Pielou’s evenness and *Bacteroidota*, restoring the microbiota composition to a more “lean” or “healthy” state.

At the genus level, treatment of ISR after 4 weeks revealed a higher proportion of *Alistipes* compared to the NOB group, which might be a bacterial genus of particular interest in the field of obesity since *Alistipes* is a potential SCFA producer and it might play a role in reducing non-alcoholic steatohepatitis and/or liver fibrosis (53, 54).

Indeed, we observed a reduced hepatosteatosis in the OBE-ISR group with lower GLUT2, FAS, and SRBP1 (19). In addition, gut microbiota from the OBE-ISR group showed increased relative abundances of the genera *Bifidobacterium*, *Bacteroides*, *Butyricimonas*, *Lachnoclostridium*, *Flavonifractor*, *Ruminiclostridium 5*, and *Faecalibaculum*, indicating an improvement of these genera. Oligosaccharides such as FOS or inulin stimulate the growth of *Bifidobacterium* in the colon, reducing iron intake by enteric pathogens in children (55) and a rise in *Bifidobacterium* and *Bacteroides* in rats (14). It has been reported that the genus *Butyricimonas* is increased in overweight and obese individuals on a low-calorie weight loss diet with fiber content (56). *Lachnoclostridium*, a genus altered by calorie restriction (57), is more pronounced in response to non-digestible oligo- and polysaccharides in children with overweight (16). Regarding the genus *Flavonifractor*, which also exhibited higher relative abundance in the OBE-ISR group after 4 weeks of the treatment, it has been stated to be negatively correlated with body fat content and distribution in the pediatric population (58). Moreover, *Flavonifractor* genera may be associated with the lower fat deposition observed in the rats (19). Though the genus *Ruminiclostridium 5* is known to produce SCFAs in the colon (59), it has been linked to obesity and cardiometabolic traits in children with normal weight and obesity, showing that the relative abundance of *Ruminiclostridium 5* is associated with obesity and fasting plasma insulin (60). The relative abundance of genus *Faecalibaculum* is augmented in HFD feeding in mice (61) and in the galactooligosaccharides (GOS)-supplemented diet in rats (62), indicating that dietary prebiotics such as GOS may shift the gut microbiota composition to a more healthy status.

Regarding the effects on SCFAs, there was a significant increase in the levels of acetate and propionate in the ISR group, indicating a putative improvement of the intestinal barrier dysfunction and immune response (63, 64).

On the contrary, the OBE-ISR group revealed a decrease in the relative abundances of the genera *Muribaculum*, *Blautia*, and *Ruminiclostridium 9*. The results of a recent metatranscriptomic analysis of colonic microbiota are consistent with our findings; dietary fibers rich in inulin, among others, decreased *Muribaculum* genus in growing pigs (65), providing new insight into the effects of dietary fibers on animal health. There is, however, evident that the genus *Blautia* is more common in diets high in soluble fibers, which are beneficial to glucan fermentation, compared with rats fed dietary fiber derived from barley malts, brewer’s spent grain, or barley extracts (containing significant amounts of β-glucan, soluble arabinoxylan, and insoluble arabinoxylan) (66). In the case of *Ruminiclostridium 9*, a study revealed that consumption of foods containing type 2 resistant starch, in the context of HFD-fed rats, decreased the abundance of pathogen taxa associated with obesity, inflammation, and aging including *Ruminiclostridium 9*. In aged mice on HFD, modulating microbiota and metabolites improved systemic inflammation and intestinal permeability (67). Interestingly, relative abundances of genera *Tyzzerella* and *Angelakisella* augmented in the microbiota from the OBE group compared to NOB and OBE-ISR groups, establishing that the ISR group restores the abundance of these genera to the same levels as the NOB group (68).

Dietary fiber contributes to the diversity of the microbiome by providing a variety of substrates for fermentation reactions carried out by those microbes that possess the necessary enzymatic machinery to degrade these complex CHO (69). Despite the fact that dietary fibers usually drive greater species richness, it should be noted that the OBE-ISR group underwent an HFD diet, which has been linked to obesity, insulin resistance, and dyslipidemia, as well as reduced gut bacterial communities (43), including *Bacteroidota* groups (70). The Western diet or HFD has profound effects on the diversity and populations of gut microbiota, increasing the relative abundance of *Bacillota* and decreasing the abundance of *Bacteroidota* (71). Although the OBE-ISR group revealed a lower alpha diversity after the treatment, we should note that we observed an enrichment of beneficial bacteria such as *Bifidobacterium* and SCFA-producing bacteria. In line with this, it has been published that the richness of gut microbiota could be reduced in nutritional interventions based on ingredients that modulate microbiota (e.g., berberine) (72). Indeed, a lower alpha diversity does not necessarily mean better protection for the host. This is well-known in infants exclusively breastfed where a predominant bifidobacteria microbiota reduces its diversity decreasing the counts of potential bacterial pathogens. In addition, beta diversity represents how much the community changes between the groups. Weighted UniFrac takes into account the relative abundance of species/taxa shared between samples, while unweighted UniFrac only considers the presence or absence. Thus, the visualization of weighted and unweighted UniFrac distances data demonstrated that fecal samples from the OBE-ISR group at the end of 4 weeks of intervention were separated from those from NOB and OBE groups, indicating a significant shift in the relative abundance of bacteria in the OBE-ISR group after the intervention.

Beyond taxonomic composition, we conducted functional profiling of gut microbiota to identify metabolic pathways involved in the effects of ISR within an HFD feeding. This was done at the end of the treatment. In addition, we observed an increased number of bacteria associated with glycolysis, including the Entner–Doudoroff pathway, glucuronic acid degradation, D-galacturonate degradation, the super pathway of N-acetylglucosamine, N-acetylmannosamine and N-acetylneuraminate degradation, as well as PPP, indicating a higher activity of CHO metabolism pathways. As part of the metabolic pathway parallel to glycolysis known as the PPP (also referred to as phosphogluconate pathway and hexose monophosphate shunt), it maintains carbon homeostasis, provides precursors to nucleotide and amino acid biosynthesis, reduces molecules for anabolism, and combats oxidative stress (73). Likewise, the Entner–Doudoroff pathway is present in several bacteria in which it may be the main pathway of glucose catabolism under aerobic conditions and represents an offshoot of the oxidative branch of the PPP, generating NADPH from the oxidation of glucose-6-phosphate (74), and it is used in many biosynthetic reactions. We detected increased activity in the PPP, and fatty acid biosynthesis and TCA pathways in the ISR gut microbiota at the end of the treatment. This indicates that its microbiota metabolizes glucose and other sugars for energy production. The Entner–Doudoroff pathway is present in some gram-negative bacteria such as *Pseudomonas*, *Rhizobium*, and *Agrobacterium*, but not in gram-positive bacteria, with the exception of *Enterococcus faecalis* (73). In fact, we found that *Bacteroides* are negatively associated with glycolysis with the Entner–Doudoroff pathway, glucuronic acid degradation, D-galacturonate degradation, the super pathway of N-acetylglucosamine, N-acetylmannosamine and N-acetylneuraminate degradation, and PPP in the OBE group. Moreover, *Flavonifractor* was also negatively associated with D-galacturonate degradation, methanogenesis from acetate, and the fatty acid biosynthesis pathway in OBE group, and no associations were observed in either NOB or OBE-ISR group, confirming that the obesity group exhibited lower activity of these metabolic pathways.

Pellock and Redinbo (75) point out that gastrointestinal microbiota produces β-glucuronidase enzymes that remove glucuronic acid as a carbon source, as a result of which the effects of mammalian inactivation can be effectively reversed (75). Glucuronic acid enters the Entner–Doudoroff pathway and shunts the resulting pyruvate into the TCA cycle (76). The ISR exhibited increased glucuronic acid degradation pathway activity after 4 weeks, indicating an increased abundance of substrate for both the Entner–Doudoroff pathway and TCA, which exhibited higher activity in the group as was abovementioned. Similarly, D-galacturonate degradation activity is significantly higher in the OBE-ISR group. A key component of pectin D-galacturonate, which can be converted to pyruvate and glyceraldehyde-3-phosphate *via* 2-keto-3-deoxy-phosphogluconate, a characteristic intermediate in the Entner–Doudoroff pathway for sugar dissimilation (77).

N-acetylglucosamine, N-acetylmannosamine, and N-acetylneuraminate are convergent pathways for utilizing amino sugars. Both N-acetylglucosamine and N-acetylneuraminate are effective carbon sources for *Escherichia coli*, whereas N-acetylmannosamine is metabolized very slowly (78). Here, our treatment with ISR in rats fed HFD exhibits higher activity in these pathways compared to OBE and NOB groups, which may help to maintain the barrier function in the intestine (79).

On the contrary, key metabolic pathways such as methanogenesis, methanogenesis from acetate, and L-tyrosine degradation revealed significantly lower abundance in the ISR-treated group after 4 weeks of the treatment. D-glucarate degradation exhibited higher activity in the ISR-treated group after 4 weeks of the treatment, indicating a putative higher metabolic rate in the CHO metabolism. Of interest, D-glucarate is an effective antitumoral agent able to bind to environmental carcinogens like benzo[a]pyrene (80, 81). Methanogenesis pathway promotes the removal of gaseous fermentation by-products, carbon dioxide and hydrogen, from the distal gut ecosystem converting carbon dioxide plus hydrogen to methane by methanogenic Archaea (82, 83). Rather than host absorption, these substrates are utilized primarily by cross-feeding between gut microbiota members (84).

Bacteria degrade L-tyrosine mainly through the production of phenolic compounds. Here, ISR treatment decreased significantly the L-tyrosine degradation pathway in the context of HFD feeding in rats. A major microbial metabolite derived from tyrosine is phenol (phenol and p-cresol) (85). A high level of phenol is associated with chronic kidney disease, cardiovascular disease, and altered cellular immune responses (86). Recent research has identified 36 phenol-producing bacteria and 55 p-cresol-producing bacteria; strong phenol producers were found in the *Coriobacteriaceae*, *Enterobacteriaceae*, *Fusobacteriaceae*, and *Clostridium* clusters I and XIVa, and strong p-cresol producers were found in the *Coriobacteriaceae* and *Clostridium* clusters XI and XIVa (87). As a result, ISR could contribute to a reduction in the production of phenols by inhibiting the degradation of L-tyrosine and assist in identifying the relationship between microbiota and host disease.

In hepatocytes, primary bile acids, cholic and chenodeoxycholic acids, are synthesized from cholesterol. Those acids are normally coupled with glycine to produce glycocholic acid and glycochenodeoxycholic acid, and taurine to produce tauro-conjugates (37). The total pool of conjugated bile acids is dominated by glycine-conjugated bile acids (88). These acids are collected in the gallbladder and liberated into the intestine to facilitate absorption of dietary lipids and liposoluble vitamins, protection against bacterial overgrowth, and elimination of cholesterol from the body (88). In the distal ileum, approximately 95% of the bile acids are reabsorbed and return to the liver through the enterohepatic circulation (89). By the action of specific bacterial enzymes, the remaining 5% of primary bile acids are metabolized into the secondary bile acids, such as deoxycholic acid, lithocholic acid, and ursodeoxycholic acid (90).

Pearson’s correlations, adjusted using the FDR procedure, reported specific associations between bile acids and SCFA with microbial populations and metabolic pathways in NOB and OBE-ISR groups. However, no significant changes were observed in the OBE group. Here, we found associations of several bile acids with some phylum and some genera in the OBE-ISR group. The main genera implicated were *Butyricimonas*, *Ruminiclostridium* 5, *Faecalibaculum*, and *Bacteroidota*. In addition, butyric acid was positively associated with *Ruminiclostridium* 5. Similar results have been found previously with the same genus (91), supporting a new association between the changes in *Ruminiclostridium* 5 and the concomitant levels of butyric acid. A study in a healthy population has reported that *Butyricimonas* was correlated with a body mass index < 25 after the consumption of the Mediterranean diet. Likewise, adherence to the Mediterranean diet was associated with augmented levels of SCFAs (92). According to our study, the *Butyricimonas* genus is related to the bile acid levels, and according to recent reports, the genus is related to the body mass index in healthy volunteers. These associations in the OBE-ISR group between *Butyricimonas* and *Ruminiclostridium* 5 should be highlighted. This is because the NOB group showed a significant association between body weight and the same bacterial genera.

There are two dominant phyla in the gut microbiota, *Bacillota* and *Bacteroidota* (93). There is an opposite trend in the abundance of *Bacteroidota* with the elderly having an abundance 80% lower than children (94). The levels of *Bacteroidota* were significantly elevated in the ISR group, and they were positively associated with taurocholic and taurodeoxycholic acids.

In the case of GLP-1, we previously reported augmented levels for the OBE-ISR group (19), and in our study, we have found a negative association with *Bacteroides*, which are prevalent in the gut of individuals living with high-fat and protein diets (95, 96). It appears that *Bacteroides* spp. play a key role in the immunomodulation of the human immune system (96). In our study, *Bacteroides* levels are elevated at the end of the intervention in the OBE-ISR group, which may be related to GLP-1 secretion, which in turn regulates insulin secretion, appetite, and body weight (97–100).

The number of basic studies addressing the analysis of microbial, anthropometric, metabolic, and inflammation data is not enough. The need for general view analyses is currently an imperative issue in the nutrition field. Consequently, recent studies using a similar methodology have reported an increase in beneficial bacteria in the colonic microbiota, as well as increased activity of enzymes involved in liver lipid metabolism (101). Further studies with similar and more advanced methodologies are necessary.

## Conclusion

**FIGURE 5:**
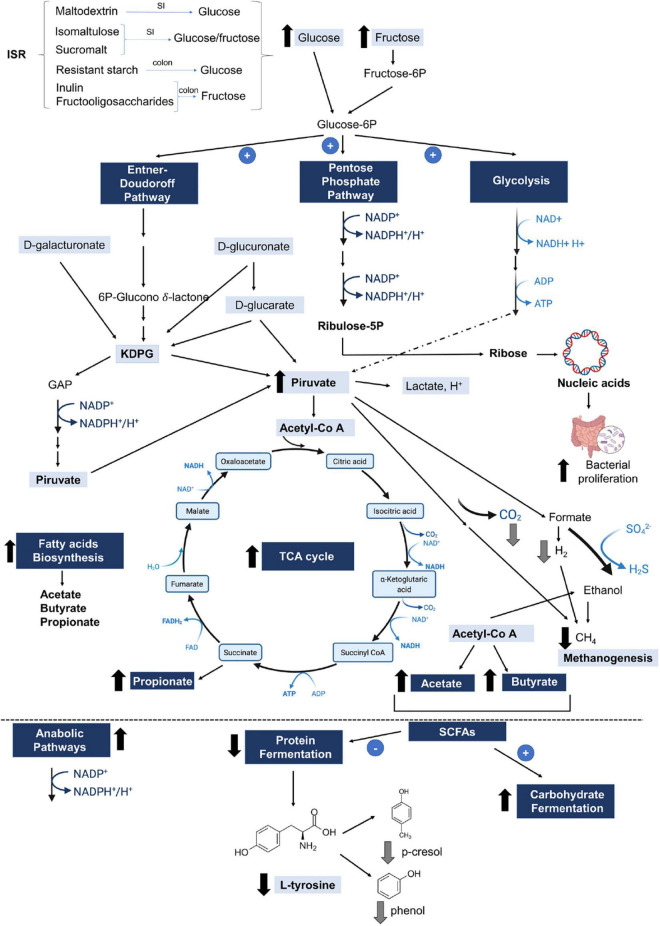
Simplified diagram of the main microbial metabolic pathways affected by HFD-ISR diet in the context of HFD. ISR containing isomaltulose, sucromalt, maltodextrin, resistant starch, inulin, and fructooligosaccharides rises glucose and fructose in the intestine, which it promotes the Entner–Doudoroff pathway, and pentose phosphate and glycolysis pathways by gut microbiota, increasing the pyruvate production and bacterial proliferation. Pyruvate increases the short-chain fatty acid (SCFA) production (butyrate, acetate, and propionate) and decreases the methanogenesis. Hence, the elevated SCFA promotes the carbohydrates fermentation and decreases the protein fermentation, lowering the L-tyrosine production, which may potentially reduce the p-cresol and phenol in the intestine. 2-keto-3-deoxy-phosphogluconate (KDPG); glyceraldehyde 3-phosphate (GAP); tricarboxylic acid (TCA) cycle. Black arrows represent the analyzed metabolic pathways. Gray arrows represent the putative products affected by the metabolic pathways according to the literature. SI, Small intestine.

In conclusion, we show that consumption of ISR within an HFD feeding in growing rats as a model of childhood obesity modifies the gut microbiota composition and provokes a shift in the functional pathway profile, which might have a protective effect against the development of metabolic disturbances in obesity (Figure 5). In addition, the increase in SCFAs resulting from ISR seems to be of relevance, since SCFAs have been shown to contribute to dietary energy harvest, modulate host adiposity, and alter the expression of host satiety hormones (102, 103). All together our results suggest that the ISR mix could preserve the gut microbiota from the dysbiosis resulting from obesity during childhood.

## Data availability statement

The data presented in this study are deposited in the National Center for Biotechnology Information (NCBI) repository, accession number Bioproject PRJNA866156.

## Ethics statement

The animal study was reviewed and approved by the animal study was reviewed and approved by the Consejería de Agricultura, Ganaderia, Pesca y Desarrollo Sostenible, Junta de Andalucia, Spain.

## Author contributions

MM, MTG-C, RR, and JML-P did the conceptualization and supervised the data. MM, RS, MDG, and ÁG carried out the data curation and investigated the data. JP-D, FR-O, AS-F, and MTG-C carried out the funding acquisition. RS, MDG, JML-P, and MM wrote the original draft. RS, MDG, RR, MM, JML-P, JP-D, FR-O, and ÁG wrote, reviewed, and edited the manuscript. All authors have read and agreed to the published version of the manuscript.
