# Supplementary material for: Intake of slow-digesting carbohydrates is related to changes in the microbiome and its functional pathways in growing rats with obesity induced by diet
Source: Front Nutr. 2022 Nov 30;9:992682. doi: 10.3389/fnut.2022.992682 (PMC9748084; doi:10.3389/fnut.2022.992682)
Supplement: Supplementary file 1 [file Table_1.DOCX]

**Supplementary Table 1.** Pearson correlations for the NOB group at the end of the intervention.

| Variables (NOB group) | **GLP-1 pmol/L** | **Leptin pg/mL** | **IL1beta pg/mL** | **Methanogenesis** | **L-tyrosine degradation** | ***Bacteroidota*** | ***Bacillota*** |
| --- | --- | --- | --- | --- | --- | --- | --- |
| Body weight | - | - | - | - | - | - | - |
| HOMA-IR index | - | - | - | - | - | - | 0.907* |
| Cholic acid | - | -0.958** | - | - | - | - | - |
| Chenodeoxycholic acid | - | -0.917** | - | - | - | - | - |
| Ursodeoxycholic + Hyodeoxycholic acid | - | -0.92** | - | - | - | - | - |
| Glycocholic acid | - | - | -0.915* | - | - | - | - |
| Glycochenodeoxycholic acid | - | - | -0.827* | - | - | - | - |
| Taurocholic acid | - | - | - | - | - | - | - |
| Taurodeoxycholic acid | - | - | - | 0.763* | - | - | - |
| TG(50:1) | - | - | - | - | - | -0.89* | - |
| PE(16:0/20:4) | -0.822* | - | - | - | - | - | - |
| Acetic acid | - | - | - | -0.998** | -0.957* | - | - |
| Propionic acid | - | - | - | -0.97** | -0.926* | - | - |
| GLP-1 pmol/L | - | - | - | - | - | - | - |
| IL1beta pg/mL | - | - | - | - | - | - | - |
| MCP-1 pg/mL | - | - | - | - | 0.799* | - | - |
| Glycolysis with Entner-Doudoroff | - | - | - | - | - | - | - |
| Glucoronic acid degradation | - | - | - | - | - | - | - |
| Galacturonate degradation | - | - | - | - | - | - | - |
| N-actyl-glucosamine, mannosamine and neuroaminate degradation | - | - | - | - | - | - | - |
| Pentose phosphate pathway | - | - | - | - | - | - | - |
| Methanogenesis | - | - | - | - | - | - | - |
| Methanogenesis from acetate | - | - | - | - | - | - | - |
| Fatty acid biosynthesis | - | - | - | - | - | - | - |
| Tricarboxylic acid cycle (prokaryotic) | - | - | - | - | - | - | - |
| D-glucarate degradation | - | - | - | - | - | - | - |

**Supplementary Table 1.** Continued

| Variables (NOB group) | ***Bifidobacterium*** | ***Bacteroides*** | ***Blautia*** | ***Butyricimonas*** | ***Muribaculum*** | ***Rikenellaceae*  RC9 gut group** | ***Flavonifractor*** | ***Ruminiclostridium* 5** |
| --- | --- | --- | --- | --- | --- | --- | --- | --- |
| Body weight | - | - | - | -0.844* | - | - | - | -0.828* |
| HOMA-IR index | - | - | - | - | - | - | - | - |
| Cholic acid | - | - | - | - | - | - | - | - |
| Chenodeoxycholic acid | - | - | - | - | 0.819* | - | - | - |
| Ursodeoxycholic + Hyodeoxycholic acid | - | - | - | - | - | - | - | - |
| Glycocholic acid | 0.883* | - | - | - | - | - | - | - |
| Glycochenodeoxycholic acid | - | - | -0.962* | - | - | - | - | - |
| Taurocholic acid | - | - | - | - | - | - | - | 0.857* |
| Taurodeoxycholic acid | - | - | - | - | - | - | - | 0.873* |
| TG(50:1) | - | - | - | - | - | - | - | - |
| PE(16:0/20:4) | - | - | - | - | - | -0.83* | - | - |
| Acetic acid | - | - | - | 0.999** | - | - | - | - |
| Propionic acid | - | - | - | - | - | - | - | - |
| GLP-1 pmol/L | - | - | - | - | - | 0.837* | - | - |
| IL1beta pg/mL | - | - | 0.862* | - | - | - | - | - |
| MCP-1 pg/mL | - | - | - | - | - | - | - | - |
| Glycolysis with Entner-Doudoroff | - | -0.875* | - | - | - | - | - | - |
| Glucoronic acid degradation | - | -0.915* | - | - | - | - | - | - |
| Galacturonate degradation | - | -0.887* | - | - | - | - | -0.839* | - |
| N-actyl glucosamine, mannosamine and neuroaminate degradation | - | -0.887* | - | - | - | - | - | - |
| Pentose phosphate pathway | - | -0.905* | - | - | - | - | - | - |
| Methanogenesis | - | - | - | 0.924* | - | - | - | - |
| Methanogenesis from acetate | - | -0.888* | - | - | - | - | -0.819* | - |
| Fatty acid biosynthesis | - | - | - | - | - | - | -0.817* | - |
| Tricarboxylic acid cycle (prokaryotic) | - | - | - | - | - | - | -0.827* | - |
| D-glucarate degradation | - | -0.817* | - | - | - | - | -0.825* | - |

**Abbreviations**, HOMA-IR, Homeostatic Model Assessment for Insulin Resistance; GLP-1, Glucagon-like peptide-1; IL, interleukin; MCP-1, Monocyte chemoattractant protein-1, TG(50:1), glycerolipids. Subclass: Triacylglycerols. Composition: TG(16:0+18:1+16:0); PE(16:0/20:4), glycerophospholipids. Subclass: 1-ether, 2-acylglycerophosphoethanolamine). **p* <0.05, ***p* <0.001.

**Supplementary Table 2.** Pearson correlations for the OBE group at the end of the intervention.

| Variables (OBE group) | **GLP-1 pmol/L** | **D-glucarate degradation** | ***Butyricimonas*** |
| --- | --- | --- | --- |
| Taurodeoxycholic acid | -0.304* | - | -0.755* |
| GLP-1 pmol/L | - | - | 0.772* |
| *Blautia* | - | -0.711* | - |

**Abbreviations**, GLP-1, Glucagon-like peptide-1. **p* <0.05.

**Supplementary Table 3.** Pearson correlations for the ISR group at the end of the intervention.

| Variables (ISR group) | **Glucoronic acid degradation** | **PPP** | **MTG** | **MTG from acetate** | **L-tyrosine  degradation** | **Tricarboxylic acid cycle (prokaryotic)** | ***Bacteroidota*** |
| --- | --- | --- | --- | --- | --- | --- | --- |
| Deoxycholic acid | - | - | - | - | - | - | - |
| Cholic acid | 0.657* | - | - | - | - | - | - |
| Chenodeoxycholic acid | 0.739* | 0.639* | - | - | - | - | - |
| Ursodeoxycholic + Hyodeoxycholic  acid | 0.755* | - | - | - | - | - | 0.665* |
| Glycocholic acid | - | - | 0.878** | 0.759* | 0.729* | - | - |
| Glycochenodeoxycholic acid | - | - | 0.681* | - | - | - | - |
| Taurocholic acid | - | - | - | - | - | - | 0.72* |
| Taurodeoxycholic acid | 0.704* | - | - | - | - | - | 0.728* |
| PE(16:0/20:4) | - | - | - | - | - | - | - |
| Acetic acid | - | - | - | - | - | -0.693* | - |
| Propionic acid | - | - | - | - | - | -0.677* | - |
| Butiric acid | - | - | - | - | - | - | - |
| GLP-1 pmol/L | - | - | - | - | - | - | - |
| IL1ß pg/mL | - | - | - | - | - | - | - |
| TNFα pg/mL | - | - | - | - | - | - | - |
| Galacturonate degradation | - | - | - | - | - | - | - |
| N-actyl- glucosamine, mannosamine and  neuroaminate degradation | - | - | - | - | - | - | - |
| L-tyrosine degradation | - | - | - | - | - | - | - |

**Supplementary Table 3.** Continued.

| Variables (ISR group) | ***Bacillota*** | ***Bifidobacterium*** | ***Bacteroides*** | ***Butyricimonas*** | ***Muribaculum*** | ***Flavonifractor*** | ***Rumini***  ***clostridium* 5** | ***Rumini***  ***clostridium* 9** | ***Faecali baculum*** |
| --- | --- | --- | --- | --- | --- | --- | --- | --- | --- |
| Deoxycholic acid | - | - | - | 0.969* | - | - | 0.98 | - | 0.973* |
| Cholic acid | - | - | - | - | - | - | - | - | 0.656* |
| Chenodeoxycholic acid | - | - | - | - | 0.698* | - | - | - | - |
| Ursodeoxycholic + Hyodeoxycholic  acid | - | - | - | - | 0.686* | - | - | - | 0.668* |
| Glycocholic acid | - | - | - | - | - | - | - | - | - |
| Glycochenodeoxycholic acid | - | - | - | - | - | - | - | - | - |
| Taurocholic acid | - | - | - | - | - | - | - | - | - |
| Taurodeoxycholic acid | - | - | - | - | - | - | - | - | - |
| PE(16:0/20:4) | - | -0.654* | - | -0.733* | - | - | - | - | -0.74* |
| Acetic acid | - | - | - | - | - | - | - | - | - |
| Propionic acid | - | - | - | - | - | - | - | - | - |
| Butiric acid | - | - | - | - | - | - | 0.821* | - | - |
| GLP-1 pmol/L | - | - | -0.711* | - | - | - | - | - | - |
| IL1ß pg/mL | - | - | - | - | - | - | 0.678* | - | - |
| TNFα pg/mL | - | - | - | - | - | - | 0.731* | - | - |
| Galacturonate degradation | 0.678* | - | - | - | - | - | - | - | - |
| N-actyl-glucosamine, mannosamine and  neuroaminate degradation | 0.708* | - | - | - | - | - | - | 0.641* | - |
| L-tyrosine degradation | - | - | - | - | - | -0.636* | - | - | - |

**Abbreviations**, GLP-1, Glucagon-like peptide-1; IL, interleukin; PE(16:0/20:4), glycerophospholipids. Subclass: 1-ether, 2-acylglycerophosphoethanolamine); TNFα, tumor necrosis factor alpha; PPP, pentose phosphate pathway, MTG, methanogenesis. **p* <0.05, ***p* <0.001.
